# Supplementary figures and images for: Rvb1/Rvb2 proteins couple transcription and translation during glucose starvation
Source: eLife. 2022 Sep 15;11:e76965. doi: 10.7554/eLife.76965 (PMC9531950; doi:10.7554/eLife.76965)

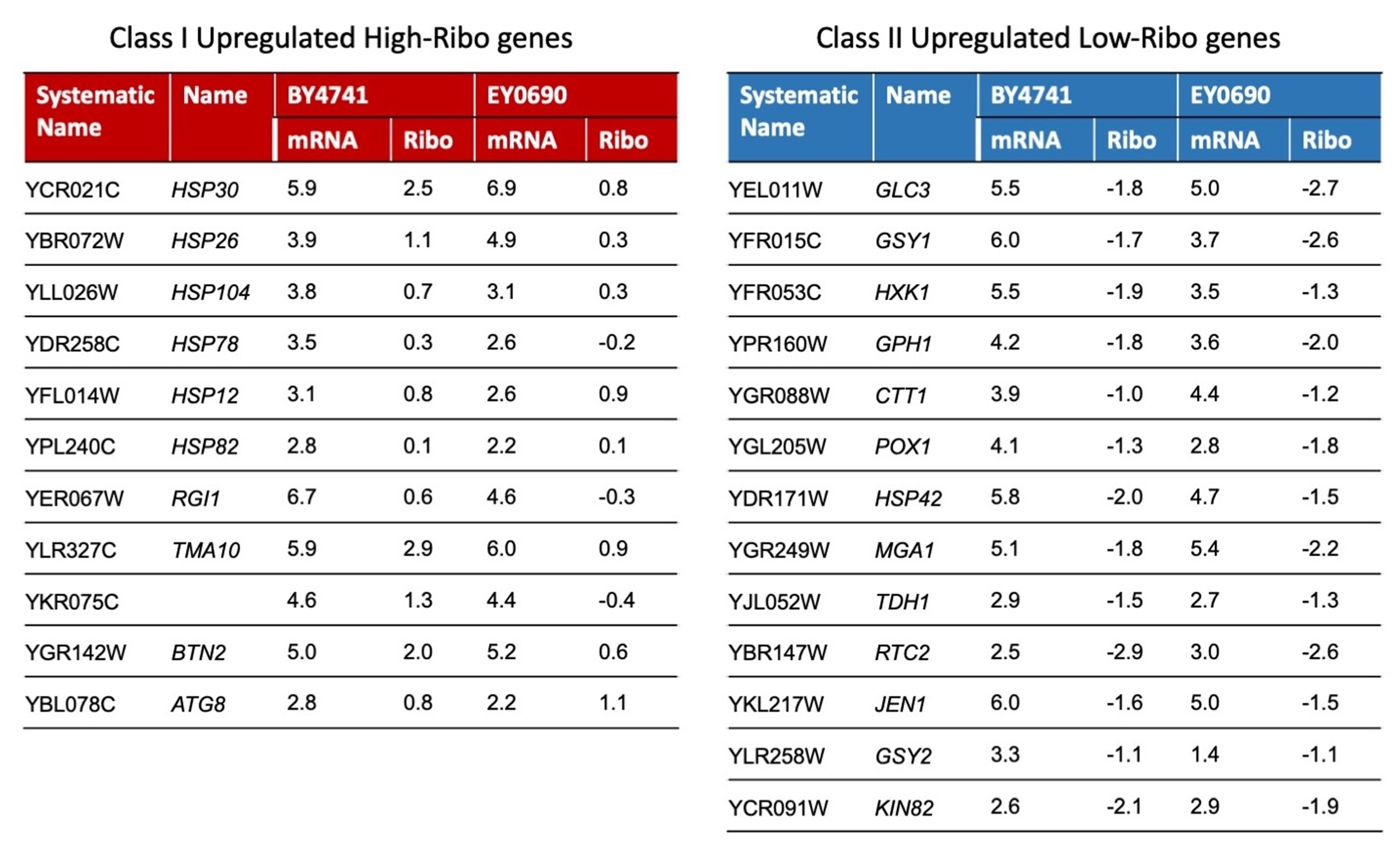

Supplement: Figure 2—source data 1. — Data from Zid and O’Shea, 2014. Fold change in mRNA levels and in ribosome occupancy after 15 min of glucose starvation from one measurement of BY4741 and one measurement of EY0690. mRNA: log2 mRNA fold change for glucose starvation vs. log-phase glucose-rich. Ribo: log2 ribosome occupancy fold change for glucose starvation vs. log-phase glucose-rich. [file elife-76965-fig2-data1.zip › Figure_2figure_supplement_2.tif]

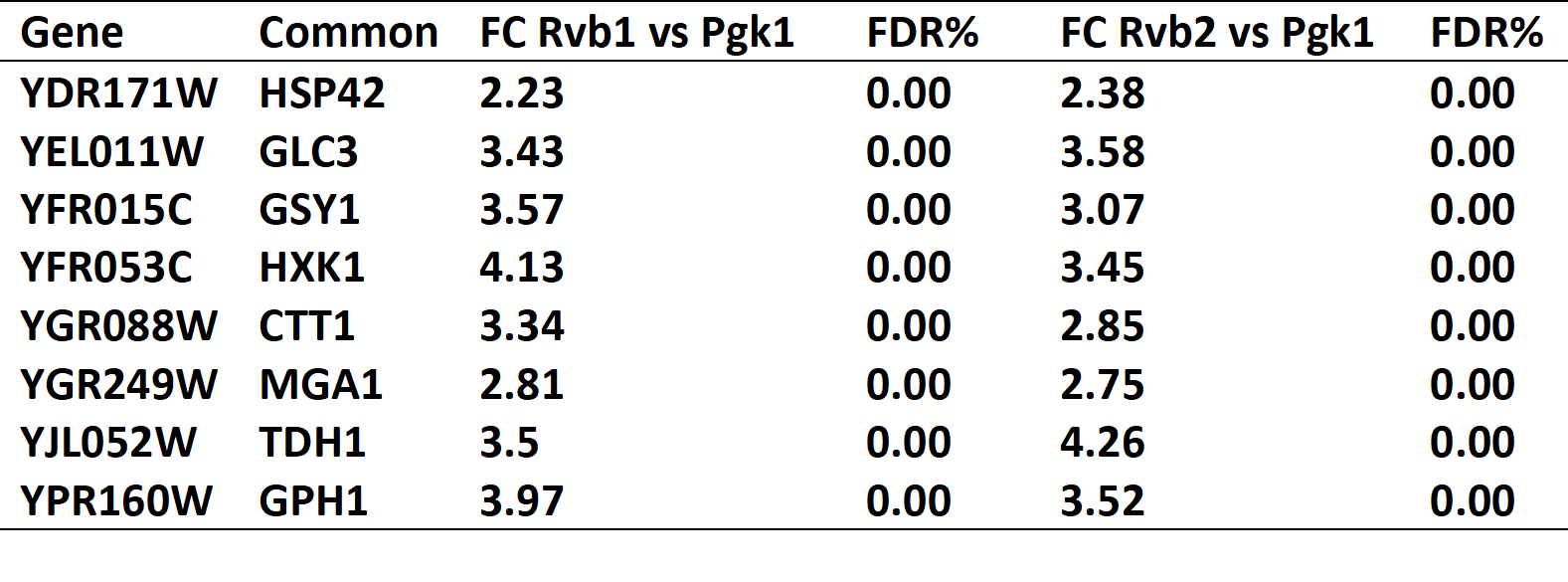

Supplement: Figure 2—source data 2. — MACS algorithm was applied from the Chromatin ImmunoPrecipitation sequencing (ChIP-seq) results of Rvb1/Rvb2 in 10 min glucose starvation. Genes are shown under systematic names. FC: fold change of Rvb’s peak versus Pgk1’s peak; FDR: false discovery rate. [file elife-76965-fig2-data2.zip › Figure_2figure_supplement_3.tif]
